# Guinea Fowl Coronavirus Diversity Has Phenotypic Consequences for Glycan and Tissue Binding

**DOI:** 10.1128/JVI.00067-19

**Published:** 2019-05-01

**Authors:** Kim M. Bouwman, Mattias Delpont, Frederik Broszeit, Renaud Berger, Erik A. W. S. Weerts, Marie-Noëlle Lucas, Maxence Delverdier, Sakhia Belkasmi, Andreas Papanikolaou, Geert-Jan Boons, Jean-Luc Guérin, Robert P. de Vries, Mariette F. Ducatez, Monique H. Verheije

**Affiliations:** a Department of Pathobiology, Faculty of Veterinary Medicine, Utrecht University, Utrecht, The Netherlands; b IHAP, Université de Toulouse, INRA, ENVT, Toulouse, France; c Department of Chemical Biology and Drug Discovery, Utrecht Institute for Pharmaceutical Sciences, Utrecht University, Utrecht, The Netherlands; Loyola University Chicago

**Keywords:** coronavirus, glycan receptor, guinea fowl, receptor affinity, spike protein, tissue tropism

## Abstract

Guinea fowl coronavirus (GfCoV) causes fulminating enteritis that can result in a daily death rate of 20% in guinea fowl flocks. Here, we studied GfCoV diversity and evaluated its phenotypic consequences. Over the period of 2014 to 2016, affected guinea fowl flocks were sampled in France, and avian coronavirus presence was confirmed by PCR on intestinal content and immunohistochemistry of intestinal tissue. Sequencing revealed 89% amino acid identity between the viral attachment protein S1 of GfCoV/2014 and that of the previously identified GfCoV/2011. To study the receptor interactions as a determinant for tropism and pathogenicity, recombinant S1 proteins were produced and analyzed by glycan and tissue arrays. Glycan array analysis revealed that, in addition to the previously elucidated biantennary di-*N*-acetyllactosamine (diLacNAc) receptor, viral attachment S1 proteins from GfCoV/2014 and GfCoV/2011 can bind to glycans capped with alpha-2,6-linked sialic acids. Interestingly, recombinant GfCoV/2014 S1 has an increased affinity for these glycans compared to that of GfCoV/2011 S1, which was in agreement with the increased avidity of GfCoV/2014 S1 for gastrointestinal tract tissues. Enzymatic removal of receptors from tissues before application of spike proteins confirmed the specificity of S1 tissue binding. Overall, we demonstrate that diversity in GfCoV S1 proteins results in differences in glycan and tissue binding properties.

**IMPORTANCE** Avian coronaviruses cause major global problems in the poultry industry. As causative agents of huge economic losses, the detection and understanding of the molecular determinants of viral tropism are of ultimate importance. Here, we set out to study those parameters and obtained in-depth insight into the virus-host interactions of guinea fowl coronavirus (GfCoV). Our data indicate that diversity in GfCoV viral attachment proteins results in differences in degrees of affinity for glycan receptors, as well as altered avidity for intestinal tract tissues, which might have consequences for GfCoV tissue tropism and pathogenesis in guinea fowls.

## Abstract (precis)

Avian coronaviruses cause major global problems in the poultry industry. As causative agents of huge economic losses, the detection and understanding of the molecular determinants of viral tropism are of ultimate importance. Here, we set out to study those parameters and obtained in-depth insight into the virus-host interactions of guinea fowl coronavirus (GfCoV). Our data indicate that diversity in GfCoV viral attachment proteins results in differences in degrees of affinity for glycan receptors, as well as altered avidity for intestinal tract tissues, which might have consequences for GfCoV tissue tropism and pathogenesis in guinea fowls.

## INTRODUCTION

Avian coronaviruses (AvCoVs) pose a major threat to poultry health, production, and welfare worldwide. AvCoVs are highly infectious, remain endemic in poultry populations, and, due to their high mutation rate, frequently produce new antigenic variants (1, 2). The best-known AvCoV is infectious bronchitis virus (IBV), causing mainly respiratory disease in chickens. In addition, IBV-like viruses have been detected in other domestic poultry, including turkey and quail (3–5). In guinea fowl, coronaviruses were identified for the first time in 2011 as the causative agent for fulminating enteritis (6). Full-genome sequencing revealed that guinea fowl coronavirus GfCoV/FR/2011 is closely associated with turkey coronavirus (TCoV) (7), with both causing gastrointestinal tract infections in their respective hosts (6, 8). Clinical signs related to GfCoV infection in guinea fowl include prostration, ruffled feathers, and decreased water and feed consumption, and GfCoV infection has resulted in a daily death rate of up to 20% in several farms in France (6). Upon necropsy of affected animals, whitish and enlarged pancreases were consistently reported. Histopathological analyses revealed pancreatic necrosis and lesions of various intensities in the intestinal epithelium, with the most severe lesions found in the duodenum of affected animals (6).

Genetic classification of AvCoVs is based on phylogenetic analysis of the S1 domain of its viral attachment protein spike (2). The spike protein is the main determinant for tropism (9), and the N-terminal part of the S1 of IBV has been shown to contain the receptor-binding domain (RBD) (10). Studies using recombinant IBV S1 and/or RBD proteins have demonstrated that the viral tropism is reflected by tissue binding of such proteins (11). Mutations in the spike proteins of IBV might result in either decreased (10) or increased (12) avidity for its receptor present on epithelial cells of the chicken trachea. In contrast to IBV, GfCoV and TCoV target the epithelial cells of the gastrointestinal tract (4, 6), and recombinant protein binding of their S1 proteins reflects this viral tropism, with predominant staining of epithelial cells of the small intestine (4). Glycan array analysis identified elongated biantennary *N*-acetyllactosamine (di-LacNAc) molecules on N-glycans as the host receptor for enteric AvCoVs, which are abundantly expressed on intestinal tissues (4).

Clinical symptoms in guinea fowl similar to those reported in 2011 are continuously reported by veterinarians in France (personal communication). However, studies on newly emerging GfCoVs are particularly hampered by the lack of models to grow the virus. More specifically, susceptible cell lines have not yet been identified, inoculation of embryonated guinea fowl eggs did not result in GfCoV production (data not shown), and specific-pathogen-free (SPF) guinea fowls are not available for experimental infection.

Here, we set out to study the consequences of GfCoV genetic diversity for glycan and tissue interactions. We revealed that the GfCoV spike gene from the 2014–2016 outbreak in guinea fowl flocks in France was 89% identical to that of GfCoV/2011 (7). Glycan and tissue binding analyses of GfCoV/2011 and GfCoV/2014 recombinant spike S1 proteins revealed that while both proteins had the same specificities, GfCoV/2014 S1 had a much higher affinity toward glycan receptors and tissues of the lower gastrointestinal tract, in agreement with the observed replication of the virus in these tissues from field cases. Taking these observations together, we demonstrate that GfCoV diversity results in phenotypically different receptor binding properties.

## RESULTS

### Lesions and coronaviral protein expression in the gastrointestinal tract of diseased guinea fowls between 2014 and 2016.

**FIG 1:**
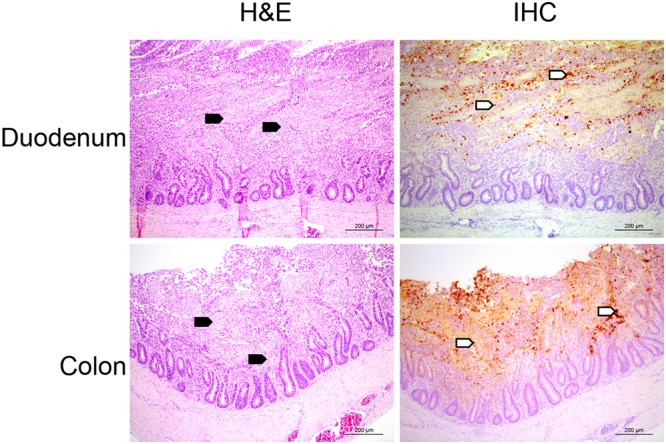
(Immuno)histological analyses of guinea fowl intestinal tract. Shown are representative images of duodenum and colon from a guinea fowl presenting with peracute enteritis in 2014 after samples were stained with H&E or antibodies against the M protein of infectious bronchitis virus, known to cross-react with GfCoV M protein in immunohistochemistry (IHC). Black arrowheads indicate inflammatory cells, and white arrowheads indicate viral protein expression.

Fulminating disease (peracute enteritis) in guinea flocks continued to be reported after the initial outbreak of GfCoV infection in 2011 (6). Between February 2014 and November 2016, duodena from 29 diseased guinea fowls were collected and analyzed for lesions and coronaviral protein expression. Histological analysis of tissues by hematoxylin and eosin (H&E) staining revealed lesions in all duodena, with clear infiltration of inflammatory cells in remnants of the villi (Fig. 1, black arrowheads). For seven animals, the entire gastrointestinal tract was available for histological analysis, showing lesions across the entire length of the intestinal tract, including the colon (Fig. 1, black arrowheads). Viral protein expression using antibodies against the M protein of avian coronaviruses was observed in all duodena and in four out of the seven lower intestinal tracts by immunohistochemistry (IHC) (Fig. 1, white arrowheads). In the colons devoid of expression of viral proteins, the infiltration of inflammatory cells was noted, suggestive of a previous exposure to a virologic agent.

In contrast to what we observed, virus replication of GfCoV/2011 appeared to be restricted to the duodenum (6). Unfortunately, we were unable to confirm the lack of infection of lower gastrointestinal tract samples in the previous outbreak due to unavailability of samples. Nevertheless, we here hypothesize that genetically divergent GfCoVs might have caused phenotypic differences in guinea fowls over the years.

### Circulation of genetically diverse GfCoV.

Gastrointestinal content collected from 20 affected animals between February 2014 and November 2016 was analyzed for the presence of gammacoronavirus genetic material by one-step real-time reverse transcription-PCR (RT-PCR) using pan-gammacoronavirus primers (13). For all samples, threshold cycle (*C_T_*) values obtained were below 35 (data not shown), confirming the presence of coronaviral RNA in all tested samples (Table 1). Next, overlapping conventional PCRs were performed with primers based on the spike gene of the GfCoV/2011 virus (sequences available upon request). Partial S1 sequences could be obtained from 10 of 20 RT-PCR-positive samples (893 to 1,841 nucleotides [nt]; 3,624 nt in the complete S sequence) (Table 1); the quality and/or quantity of the remaining 10 samples was too low to generate PCR products. Sanger sequencing of the obtained fragments confirmed the presence of GfCoV in the intestinal content of all 10 birds, confirming continuous GfCoV circulation in France.

**TABLE 1:** Overview of selected guinea fowls and obtained GfCoV spike sequences

Animal no.^*a*^	Date of sample collection (wk no./yr)	Age at sampling time (wk)^*b*^	GenBank accession no. (spike sequence [nt])	% nucleotide identity with GfCoV/2011 S1^*c*^
14-002	6/2014	10		
14-013	15/2014	8		
**14-032**	22/2014	7	MG765535 (1–3669)	85
**14-036**	24/2014	7		
14-037	25/2014	7		
14-039	26/2014	5.5		
14-040	23/2014	ND	MG765536 (1–1392)	88
14-041	23/2014	ND	MG765537 (1–1771)	88
14-042	23/2014	ND	MG765538 (1–1392)	88
**14-047**	33/2014	3	MG765539 (1–1378)	88
14-053	37/2014	9	MG765540 (1–1393)	88
14-065	44/2014	12		
14-066	45/2014	4	MG765541 (1–1384)	88
**15-006**	3/2015	ND	MG765542 (1–980)	87
**15-116**	46/2015	7		
**15-118**	47/2015	8		
16-086	38/2016	ND	MK290733 (1–2465)	85
16-115	45/2016	4		
16-123	47/2016	ND	MK290734 (571–1895)	86

a Animals identified by boldface numbers were included for immunohistological examination as well.

b ND, not determined.

c Based on the S1 sequence (nt 1 to 3708) of GfCov/FR/2011, GenBank accession number LN610099.1.

**FIG 2:**
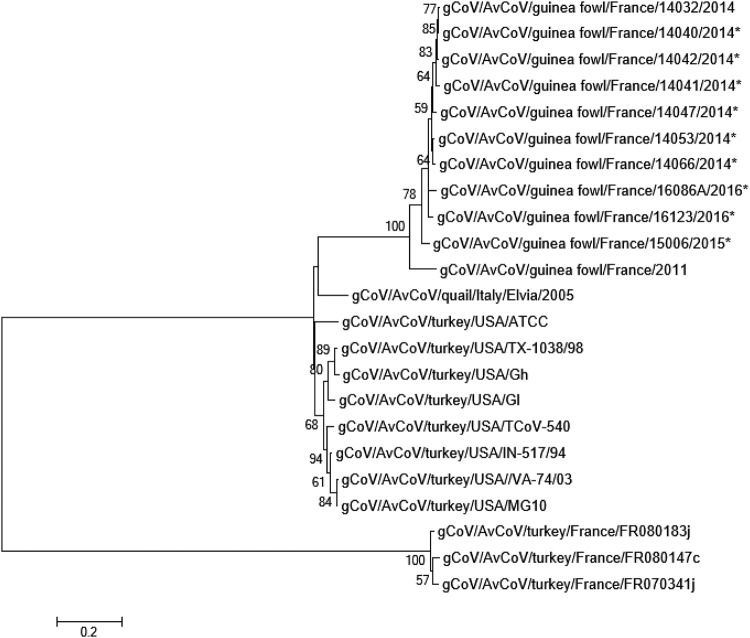
Molecular phylogenetic analysis by maximum likelihood method comparing GfCoV (partial) spike sequences. Phylogenetic tree was based on the Kimura two-parameter model, in which bootstrap values are shown next to the branches. The analysis involved 22 nucleotide sequences. All positions containing gaps and missing data were eliminated. There were a total of 893 nucleotide positions in the final data set. Evolutionary analyses were conducted in MEGA, version 6. *, partial S1 sequence of GfCoV.

Phylogenetic analysis was performed to investigate the genetic diversity of the obtained partial S1 sequences using maximum likelihood analyses (Fig. 2). The results showed that the 2014–2016 sequences clearly clustered with the S1 reference gene from GfCoV/2011 (GenBank accession number HF544506) supported by a bootstrap value of 100, while they were genetically more distantly related to TCoV. Each of the GfCoV 2014–2016 partial S1 sequences shared 85% to 88% nucleotide identity with the GfCoV/2011 S1 sequence, and between the 2014 and 2016 partial S1 sequences, the variation was 0.1 to 8.0%.

Only from one sample could a full spike sequence be obtained (γCoV/AvCoV/guinea fowl/France/14032/2014) while for the other samples the amount and/or quality of the viral RNA samples was too low for further analyses. Comparison of the S1 gene of GfCoV/2014 with that of GfCoV/2011 using the Kimura two-parameter distance model indicated that the genes had an 85% nucleotide and an 89% amino acid sequence identity. Alignment of the amino acid sequences did not indicate clear mutation hot spots (data not shown), and the huge sequence diversity with the S1 of IBV strain M41 (the only avian coronavirus for which a cryo-electron microscopy [cryo-EM] structure has been elucidated [14]) precluded further suggestions on the implications of each of the mutations.

### GfCoV/2014 S1 recognizes the enteric coronavirus diLacNAc glycan receptor with higher affinity than GfCoV/2011 S1.

**FIG 3:**
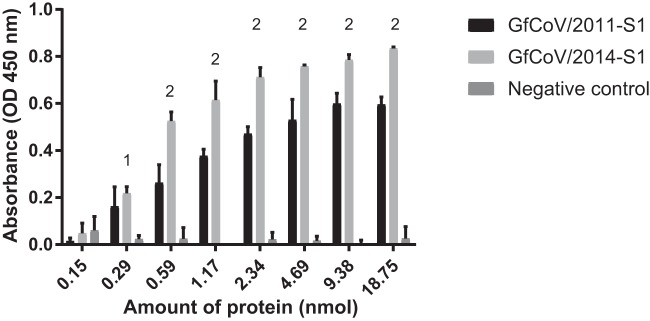
Binding of GfCoV S1 to the enteric coronavirus glycan receptor diLacNAc. Concentration-dependent binding of GfCoV S1 proteins to Galβ1,4GlcNAcβ1,3Galβ1,4GlcNAc in ELISA. The N-terminal domain of the S1 protein of IBV strain M41 was used as a negative control (10). 1, significant difference between GfCoV S1 and IBV-M41; 2, significant difference between GfCoV/2014 S1 and GfCoV/2011 S1 (*P* < 0.001).

Using the glycan array of the Consortium for Functional Glycomics, we previously determined that S1 from GfCoV/2011 specifically binds to the diLacNAc glycan receptors (Galβ1,4GlcNAcβ1,3Galβ1,4GlcNAc) (4). To study whether the observed changes in the spike of GfCoV/2014 resulted in differences in recognition of this glycan receptor, we recombinantly produced GfCoV/2014 S1 and GfCoV/2011 S1 and applied both proteins to diLacNAc-polyacrylic acid (PAA) conjugates in an enzyme-linked immunosorbent assay (ELISA) as previously described (4). At similar protein concentrations, GfCoV/2014 S1 showed improved binding to this receptor (Fig. 3), indicating that the mutations in S1 did not affect the specificity but resulted in significantly higher affinity for this particular receptor.

### The genetic differences between GfCoV/2014 and GfCoV/2011 did not alter glycan specificity.

**FIG 4:**
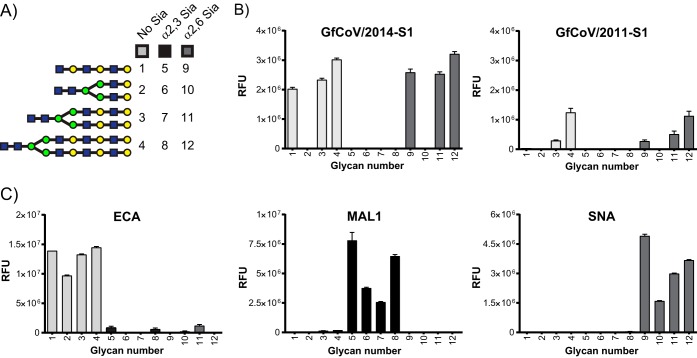
Glycan binding specificity of guinea fowl S1 proteins. (A) Schematic representation of selected glycan structures present on the glycan array; numbers correspond to those shown in the graphs. Numbers 1 to 4 represent glycans ending with galactose, numbers 5 to 8 represent glycans capped with alpha-2,3 -linked sialic acids, and numbers 9 to 12 represent glycans capped with alpha-2,6-linked sialic acids. Yellow circle, galactose; blue square, GlcNAc; green circle, mannose. (B and C) Glycan receptor specificity of GfCoV S1 proteins and lectins ECA, MAL1, and SNA in glycan array assay (Broszeit and de Vries, submitted). RFU, relative fluorescent units.

Next, we investigated whether the mutations in S1 resulted in recognition of additional N-linked glycans. To this end, both S1 proteins were applied to a novel glycan array containing N-glycan structures with their linear counterparts, either with terminal galactose or two differently linked sialic acid moieties (F. Broszeit and R. P. de Vries, submitted for publication). Schematic representations of each of the glycans are given in Fig. 4A. The data revealed that both GfCoV S1 proteins bind to longer biantennary LacNAc structures (Fig. 4B, structures 3 and 4), including the diLacNAc structure used in the ELISA (Fig. 3). Furthermore, both GfCoV S1 proteins bound to longer linear LacNAc repeats (Fig. 4B, structure 1), which were not included in the previous array (4). Finally, both GfCoV S1 proteins bound longer linear and biantennary LacNAc repeats with terminal alpha-2,6 sialic acid (Fig. 4B, structures 9 to 12) but not those capped with alpha-2,3-linked sialic acids (Fig. 4B, structures 5 to 8). Erythrina crista-galli lectin (ECA), Sambucus nigra lectin (SNA), and Maackia Amurensis lectin I (MAL1) were used as controls. We observed, as expected, specific binding to galactose and alpha-2,6-linked and alpha-2,3-linked sialic acid terminal glycans, respectively (Fig. 4C). In conclusion, both GfCoV S1 proteins show specificity for the same glycans, ending with either galactose or alpha-2,6-linked sialic acids on the glycan array. However, the relative fluorescence observed for GfCoV/2014 S1 was consistently higher than that for GfCoV/2011 S1, which is suggestive of differences in their affinities for glycan receptors, as observed for diLacNAcs in the data shown in Fig. 3.

### GfCoV/2014 S1 has higher affinity for glycan receptors than GfCoV/2011 S1.

**FIG 5:**
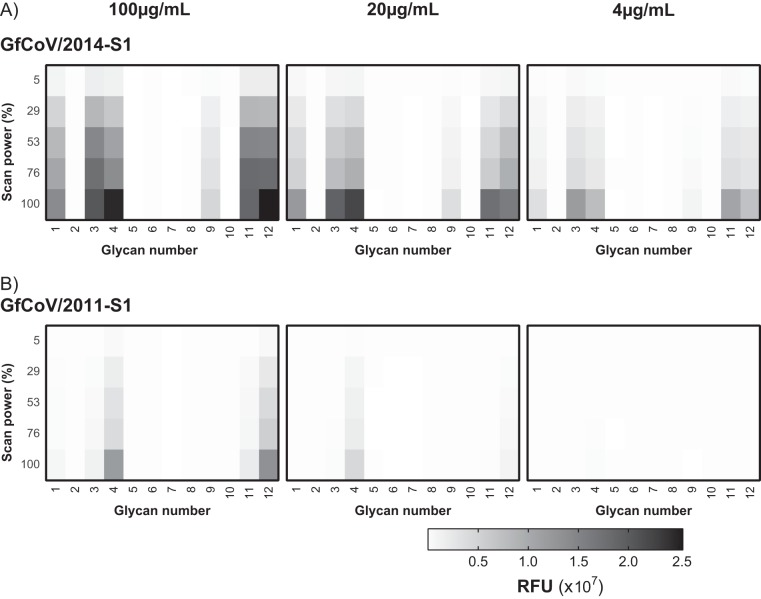
Glycan binding affinity of guinea fowl S1 proteins. (A and B) Glycan binding of GfCoV/2014 S1 and GfCoV/2011 S1 is shown as a heat map with 5-fold dilutions (100 µg/ml to 4 µg/ml) of the proteins applied to glycan array slides that were scanned at different laser intensities. RFU, relative fluorescent units. Glycan numbers correspond to schematic representations shown in Fig. 4A.

To allow comparison of the binding affinities of the two proteins for each glycan, we applied 5-fold serial S1 protein dilutions onto the glycan array and compared binding intensities at various scan powers. At each concentration, for all glycans shown in Fig. 4A, binding signals of GfCoV/2014 S1 (Fig. 5A) were consistently higher than those of GfCoV/2011 S1 (Fig. 5B). Detection of linear glycan binding (glycans 1 and 9) required higher concentrations and scan powers than the detection of biantennary LacNAc structures (glycans 3 and 4 and glycans 11 and 12) for both proteins. Interestingly, binding intensity of GfCoV/2011 S1 to glycans with terminal alpha-2,6 sialic acids was less than the binding intensity to glycans with terminal galactose (Fig. 5B, compare structures 3 and 4 to structures 11 and 12 at a protein concentration of 100 µg/ml to those at 20 µg/ml). This difference in preference for galactose-terminal glycans was not observed for GfCoV/2014 S1 since binding levels to glycan structures 3 and 4 and to structures 11 and 12 were similar in each dilution applied to the array (Fig. 5A). Taken together, the data indicate that GfCoV/2014 S1 has a higher affinity than GfCoV/2011 S1 for all glycans bound on the array.

### GfCoV/2014 S1 has broader gastrointestinal tract tropism.

To reveal whether the observed differences in glycan binding properties of the S1 proteins have biological consequences for tissue tropism, we first determined whether the identified glycans are indeed present on gastrointestinal tract tissues of healthy, uninfected guinea fowl. Both SNA and ECA lectins stained the epithelial lining of the duodenum, jejunum, and cecum intensely, while intermediate staining of the proventriculus and colon was observed. In the pancreas, only limited binding of SNA was observed, with no staining by ECA; in contrast, in the ileum ECA strongly bound whereas SNA bound only to a limited extent. In conclusion, all tissues of the gastrointestinal tract, except cloaca, express GfCoV glycan receptors (Table 2) (15).

**TABLE 2:**
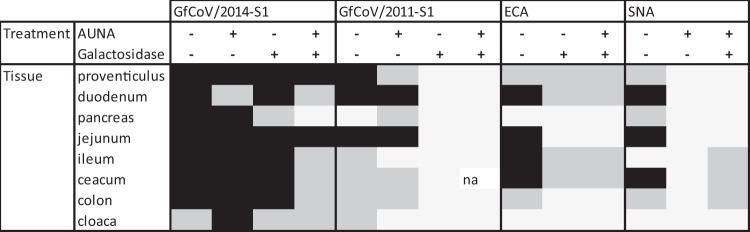
Relative binding of viral proteins and lectins on guinea fowl intestinal tissues^*a*^

a Boxes are color coded as follows: white, no visible staining; gray, light to mild staining and/or not all epithelial cells show staining; black, intense staining with most of the epithelial cells showing positive signal. na, not analyzed.

**FIG 6:**
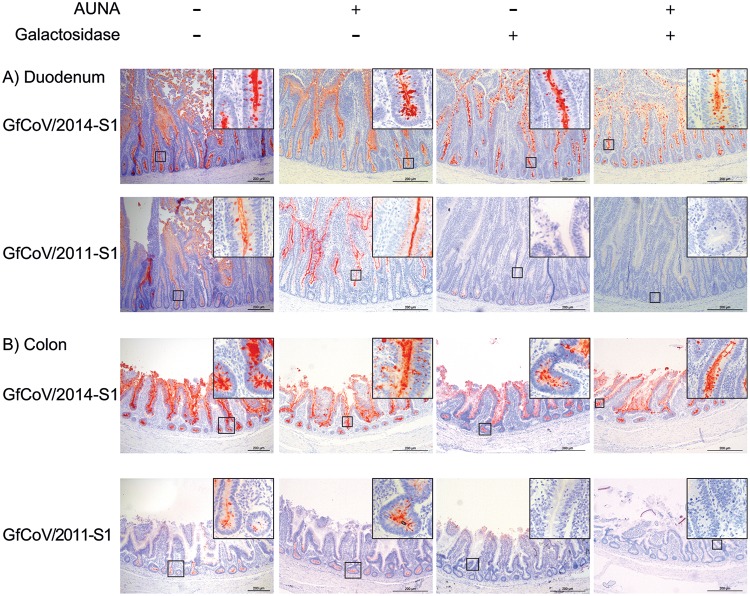
Binding of GfCoV S1 proteins to guinea fowl duodenum and colon without and with enzymatic pretreatment of the tissues. (A and B) Spike histochemistry was performed on uninfected, healthy duodenum and colon tissues without and with pretreatment of enzymes (AUNA and/or galactosidase) before application of GfCoV/2014 S1 and GfCoV/2011 S1. Binding of proteins was visualized by red staining.

Next, we investigated the binding patterns of GfCoV S1 proteins to gastrointestinal tissues. Both proteins stained the epithelial cells of almost the entire gastrointestinal tract (duodenum and colon in Fig. 6, first column; results are summarized in Table 2), indicating that receptors present on the tissues allow binding of S1. Interestingly, staining intensities of the lower intestinal tract (ileum, cecum, and colon) were much more apparent for GfCoV/2014 S1 than for GfCoV/2011 S1. This prompted us to analyze avidity and specificity of GfCoV S1 proteins to glycan receptors in the guinea fowl gastrointestinal tissues. We therefore pretreated tissue slides with Arthrobacter ureafaciens neuraminidase (AUNA) and/or galactosidase to cleave off terminal sialic acids and/or galactose residues from host glycans, respectively. Treatment of the tissues with AUNA had only a minor effect on the binding of both GfCoV S1 proteins, with a slight decrease in binding intensity to the duodenum for GfCoV/2014 S1 (Fig. 6A, second column; Table 2). SNA lectin binding was completely abolished after pretreatment with AUNA, confirming that the treatment did effectively cleave off all sialic acids from the host glycans (Table 2).

When galactose residues were removed from the tissues by treatment with galactosidase prior to application of ECA, binding was severely reduced or totally absent (Table 2). Binding of GfCoV/2011 S1 to the tissue was completely abolished (Fig. 6, third column; Table 2), indicating that GfCoV tissue engagement is almost exclusively dependent on the presence of galactose-terminating glycans. On the other hand, GfCoV/2014 S1 still clearly bound to the epithelial cells of the intestinal tract, indicating a significant difference in receptor binding avidity (Fig. 6, third column; Table 2).

Finally, tissues were simultaneously pretreated with AUNA and galactosidase to remove both galactose and sialic acids from the glycans of the host. Indeed, binding levels of both ECA and SNA were strongly reduced (Table 2). Tissue binding of GfCoV/2011 S1 was completely prevented, while GfCoV/2014 S1 still clearly bound to the epithelial cells of the gastrointestinal tract (except pancreas) (Fig. 6, fourth column; Table 2). These results suggest that either a minor amount of receptor is still present or that an additional (glycan) receptor is involved in tissue binding of GfCoV/2014 S1.

## DISCUSSION

In this study, we demonstrated ongoing GfCoV circulation in guinea fowl flocks in France. The sequence diversity between the viral attachment proteins of GfCoV circulating in 2011 and 2014 resulted in differences in receptor binding properties with profound phenotypic consequences. The relationship between these findings and *in vivo* pathogenesis can only be elucidated in detail, however, when new models to study this virus have been developed.

An amino acid sequence identity of 89% between viruses circulating only several years apart might indicate either that a novel GfCoV strain was introduced in France from a yet unidentified source or that there was high evolutionary pressure on the 2011 GfCoV strain. High mutation rates for avian coronaviruses are not uncommon (based on full-genome sequences, around 1.2 × 10^−3^ substitution/site/year [16, 17]). When the S1 sequences of GfCoV/2011 and GfCoV/2014-S1 are compared, the calculated mutation rate was 5 × 10^−2^ substitutions/site/year with a ratio of nonsynonymous to synonymous substitutions (*dN*/*dS*) of 0.45. Similar mutation rates of the spike have been reported for IBV (18) and are believed to be driven by selective pressure after vaccination (19, 20). However, no vaccine is available against GfCoV or against the closely related turkey coronavirus, TCoV. Another driver for genetic diversity is the population size (21); however, this is unlikely to explain the observed high mutation rate of GfCoV since flocks are considerably smaller than chicken flocks. It might well be that circulating antibodies against field strains of GfCoV are the main drivers of the observed sequence diversity. Unfortunately, retrospective studies to further elucidate the contribution of virus evolution, the circulation of other virus populations in the last years, or the introduction of novel strains via, for example, trade of birds between farms are impossible due to the lack of archived material.

Here, we revealed a novel glycan receptor for GfCoV, the first coronavirus that binds N-glycans capped with alpha-2,6-linked sialic acids. Alpha-2,6 sialic acid presence has been reported previously in guinea fowl large intestine (15), along with the previously elucidated poly-LacNAc expressed in guinea fowl small intestine (4). Together, their expression patterns can explain in large part the tropism of GfCoV, but this observation, combined with the results presented in the manuscript, does not exclude the possibility that yet another host factor plays a role in GfCoV/2014 infection. Initial attempts to show whether protein receptors, required for infection of many other coronaviruses (22–24), are required have yet to be successful (data not shown).

While spike protein binding analyses suggest phenotypic differences between these viruses *in vivo*, the reported gross clinical signs in field cases between 2011 and 2014 were not markedly different. Unfortunately, and in contrast to a previous study (6), attempts to study the pathogenesis of GfCoV/2014 by inoculating commercial guinea fowls with GfCoV-containing fecal samples did not result in manifestations of clinical signs or convincing detection of viral RNA by reverse transcription-quantitative PCR (RT-QPCR) (data not shown). Whether this was due to previous exposure of commercial birds to GfCoV and, hence, circulating antibodies preventing the infection remains to be investigated.

Here, we have demonstrated that GfCoV/2014 S1 has higher affinity for glycan receptors and increased avidity for the lower gastrointestinal tract than GfCoV/2011 S1. The viral genetic diversity between these spikes and the implications for receptor recognition further add to our understanding of this virus for which models are basically lacking.

## MATERIALS AND METHODS

### Collection of field samples.

Samples were collected from guinea fowls showing enteritis and concomitant high mortality (>10%) in flocks in five regions in France (Bretagne, Pays de Loire, Nouvelle-Aquitaine, Occitanie, and Auvergne-Rhône-Alpes) from February 2014 through November 2016. Gastrointestinal content was collected and stored at −80°C for viral RNA isolation. Tissues (duodenum, pancreas, air sac, lung, small intestine, large intestine, kidney, cloaca, trachea, and bursa) were collected during necropsy of euthanized or deceased animals in routine diagnostics by local veterinarians, fixed for 24 h in 4% (mass/vol) buffered formaldehyde, and stored in 70% ethanol.

### Immunohistochemistry.

Paraffin-embedded tissues were sliced at 4 µm, deparaffinized in xylene, and rehydrated in an ethanol gradient from 100% to 70%. Antigen retrieval was carried out in Tris-EDTA, pH 9.0 (preheated), before application of 1% H_2_O_2_ in methanol. After two washes in normal antibody diluent (Immunologic), the monoclonal antibody (MAb) mouse anti-IBV M protein 25.1 (Prionics, Lelystad, The Netherlands), cross-reacting with TCoV and GfCoV (5), was applied for 1 h at room temperature (RT). Slides were washed in phosphate-buffered saline (PBS)–0.1% Tween, and an EnVision kit (catalog no. K4001; Dako) was used for anti-mouse secondary antibody staining according to the manufacturer’s protocol. Slides were washed three times in PBS, and viral M protein presence was visualized with aminoethylcarbazole (AEC). The tissues were counterstained with hematoxylin and mounted with AquaMount (Merck).

### Molecular characterization of GfCoV.

The gastrointestinal content collected from affected guinea fowl was clarified by centrifugation (30 s at 11,000 × *g*), and RNA was extracted using a Qiagen Viral RNA extraction kit according to the instructions of the manufacturer. A one-step real-time RT-PCR targeting the avian coronavirus N gene was carried out to confirm the presence of coronavirus RNA as previously described (13). Subsequently, the isolated RNA was reverse transcribed using a Revertaid kit with random hexamers (Thermo Fisher, Waltham, MA), and overlapping conventional PCRs were performed to amplify the guinea fowl S gene (primer sequences available upon request). Sanger sequencing of the resulting fragments was performed using PCR primers. Contigs were generated with BioEdit (version 7.0.8.0) (25) and submitted to NCBI. MUSCLE (26) was used for the alignment, and MEGA (version 6.06) was used with a bootstrap value of 1,000 for the phylogeny (27). Selective pressure was calculated as *dN*/*dS*, and the hypothesis *dN* = *dS* was tested using the Pamilo-Bianchi-Li method (28), with a *P* value of <0.05 considered statistically significant.

### Construction of the expression vector.

The codon-optimized sequence for GfCoV/2014 S1 (γCoV/AvCoV/guinea fowl/France/14032/2014), containing upstream NheI and downstream PacI restriction sites, was obtained from GenScript and cloned into the pCD5 expression vector by restriction digestion, as previously described (29). The S1 sequence is in frame with a C-terminal GCN4 trimerization motif and Strep-tag. The expression vector encoding GfCoV/2011 S1 was generated previously (29).

### Production of recombinant proteins.

Recombinant S1 proteins were expressed by transfection of human embryonic kidney (HEK293T) cells with pCD5 expression vectors using polyethylenimine (PEI) at a 1:12 (wt/wt) ratio. Cell culture supernatants were harvested after 6 days. The recombinant proteins were purified using Strep-Tactin Sepharose beads as previously described (29).

### ELISA.

Galβ1,4GlcNAcβ1,3Galβ1,4GlcNAc (Consortium for Functional Glycomics) was coated in a 96-well Nunc MaxiSorp plate (Sigma-Aldrich) at 0.5 µg/well overnight at 4°C, followed by blocking with 3% bovine serum albumin (BSA; Sigma) in PBS–0.1% Tween. S1 proteins were preincubated with Strep-Tactin–horseradish peroxidase (HRPO) (1:200) for 30 min on ice. For each protein, 2-fold dilutions were made in triplicate in PBS and applied onto the coated well, followed by incubation for 2 h at room temperature. TMB (3,3′,5,5′-tetramethylbenzidine; Thermo Scientific) substrate was used to visualize binding, after which the reaction was terminated using 1 M H_2_SO_4_. The optical density at 450 nm (OD_450_) was measured in a FLUOstar Omega instrument (BMG Labtech), and MARS data analysis software was used for data analysis. Statistical analysis was performed using a two-way analysis of variance (ANOVA).

### Glycan array.

Glycan structures were printed in six replicates on glass slides (Nexterion Slide H; Schott Inc.). Prelabeled S1 proteins with Alexa Fluor 647-linked anti-Strep-tag mouse antibody and with Alexa Fluor 647-linked anti-mouse IgG (4:2:1 molar ratio) were applied to the slides (concentrations are given in figure legends) and incubated for 90 min, after which the slides were washed with PBS and deionized water, dried, and imaged immediately.

As controls different lectins were applied: Erythrina crista-galli agglutinin (ECA), which is specific for glycans with terminal galactose, *N*-acetylgalactosamine, or lactose, and Sambuca nigra agglutinin (SNA) and Maackia Amurensis lectin I (MAL1), which are specific for alpha-2,6-linked and alpha-2,3-linked sialic acids attached to terminal galactose, respectively. Of the six replicates, the highest and lowest values were removed, and of the remaining four replicates, the total signal values and standard deviations (SD) were calculated and plotted in bar graphs or heat maps.

### Spike histochemistry.

Spike histochemistry was performed as previously described (29). S1 proteins precomplexed with Strep-Tactin–HRPO were applied onto 4-µm sections of formalin-fixed paraffin-embedded healthy guinea fowl tissues, and binding was visualized using 3-amino-9-ethyl-carbazole (AEC; Sigma-Aldrich). Proteins were applied onto slides at 5 µg/ml. Where indicated (on Fig. 6, its legend, and the text), the tissues were treated per slide with 40 U β-galactosidase (β-Gal) (Megazyme, USA) or 2 mU of neuraminidase (sialidase) from Arthrobacter ureafaciens (AUNA) (Sigma, Germany) in 10 mM potassium acetate and 2.5 mg/ml Triton X-100, pH 4.2, and incubated at 40°C overnight (O/N) before protein application.

### Lectin histochemistry.

Lectin histochemistry was performed as previously described (4). Biotinylated Erythrina crista-galli lectin or biotinylated Sambucus nigra lectin (both from Vector Laboratories) were diluted in PBS to a final concentration of 2 µg/ml (ECA) or 6 µg/ml (SNA) and applied to healthy guinea fowl tissue sections for 30 min. After samples were washed with PBS, the signal was visualized by an avidin-biotin complex (ABC kit; Vector Laboratories) and counterstained with hematoxylin.

### Data availability.

Contigs are available in GenBank under accession numbers MG765535 to MG765542 and accession numbers MK290733 and MK290734.
